# Evaluation of strategies for the diagnosis of Chagas disease in an endemic area: SaMi-Trop study

**DOI:** 10.1590/S1678-9946202567055

**Published:** 2025-08-18

**Authors:** Ingredy Carolline de Jesus Santos, Cesar Augusto Taconeli, Ester Cerdeira Sabino, Antonio Luiz Pinho Ribeiro, Andréia Brito de Souza, Ana Beatriz Cardoso Sena, Dardiane Santos Cruz, Sâmara Fernandes Leite, Ana Clara de Jesus Santos, Amanda Mota Lacerda, Luciano de Freitas Fernandes, Desirée Sant’Ana Haikal, Israel Molina Romero, Diego Dias de Araújo, Ariela Mota Ferreira

**Affiliations:** 1 Universidade Estadual de Montes Claros, Programa de Pós-Graduação em Ciências da Saúde, Montes Claros, Minas Gerais, Brazil; 2 Universidade Federal do Paraná, Curitiba, Paraná, Brazil; 3 Universidade de São Paulo, Faculdade de Medicina, Instituto de Medicina Tropical de São Paulo, São Paulo, São Paulo, Brazil; 4 Universidade Federal de Minas Gerais, Faculdade de Medicina, Departamento de Clínica Médica, Belo Horizonte, Minas Gerais, Brazil; 5 Universidade Federal de Minas Gerais, Faculdade de Medicina, Hospital das Clínicas, Centro de Telessaúde e Serviço de Cardiologia, Belo Horizonte, Minas Gerais, Brazil; 6 Universidade Estadual de Montes Claros, Montes Claros, Minas Gerais, Brazil; 7 Fundação Oswaldo Cruz, Instituto René Rachou, Laboratório de Triatomíneos e Epidemiologia da Doença de Chagas, Belo Horizonte, Minas Gerais, Brazil; 8 Universidade Estadual de Montes Claros, Programa de Pós-Graduação em Cuidados Primários em Saúde, Montes Claros, Minas Gerais, Brazil

**Keywords:** Chagas disease, Laboratory diagnosis, Immediate tests, Primary health care

## Abstract

This study aims to evaluate strategies for diagnosing Chagas disease (CD) using rapid tests and risk questions in an endemic area. This is an analytical cross-sectional study conducted with 751 individuals from two municipalities in an endemic region for CD in the North of Minas Gerais State, Brazil. Participants answered a questionnaire with personal information and the risk criteria for CD infection recommended by the Clinical Protocol and Therapeutic Guidelines for Chagas disease (PCDT). Subsequently, they underwent capillary blood collection for the rapid diagnostic test (RDT). Individuals with a positive RDT result, along with negative controls, underwent venipuncture for serological testing. The mean age of participants was 51.4 (±18.2) years, most were female (n=434/57.8%). In the RDT, 699 (93.1%) individuals tested negative; of these, 109 (15.6%) underwent serology and 4 (3.7%) tested positive. Among the 52 (6.9%) individuals with a positive RDT result, 48 (94.1%) had their result confirmed by the serological tests. RDT result, age, and risk factors—except for blood transfusion before 1992—were statistically associated with positive serology for CD. The RDT demonstrated high sensitivity (0.92; 95% CI: 0.81–0.92) and specificity (0.97; 95%CI: 0.92–0.99).

## INTRODUCTION

Chagas disease (CD), caused by the protozoan *Trypanosoma cruzi*, is classified as a neglected disease^1,2^. Its impact extend beyond health, leading to significant socioeconomic impacts due to high rates of premature mortality, loss of productive years due to disabilities, and the substantial costs of medical care and social security support^2-4^. This makes CD a serious public health problem, affecting most Latin American countries. A meta-analysis conducted in 2014, which reviewed prevalence studies of CD in Brazil from 1980 to 2000, estimated an overall prevalence of 4.2% (95%CI: 3.1–5.7) throughout the period, with variations in the 1980s (4.4%), 1990s (7.2%), and post-2000 (2.4%)^5^. However, recent studies have reported prevalence rates up to 9.2% in Minas Gerais State, an endemic region in Brazil^6,7^.

Regarding diagnosis, during the acute phase of CD, parasitological methods can be employed due to the high concentration of parasites in the bloodstream^1^. In the chronic phase, however, because of low parasitemia, diagnosis relies on immunological methods^8^. The Pan American Health Organization (PAHO) recommends using two serological tests for patient screening^9^. This practice is not followed in the United States, where diagnostic algorithms rely on a single screening test, followed by a confirmatory test only in positive cases^10^. In blood donors, only one test is used for screening. In Brazil, however, the Clinical Protocol and Therapeutic Guidelines (PCDT) for CD still recommend combining two serological tests with different methodologies. Rapid diagnostic tests (RDTs) are suggested by the PCDT as an active case-finding strategy, especially in remote areas, in patients with limited access to healthcare services, and in pregnant women suspected of having the disease. After RDT application, serological confirmation is required. Additionally, for active case-finding, the PCDT also proposes a questionnaire to assess risk factors in the population^1^.

Despite its clinical importance and relevance, CD diagnosis remains a challenge and is impacted by its neglect, reflected in late diagnosis and missed treatment opportunities^6,7,11^. Only 10% of world population with CD have access to diagnosis^11^. Lack of knowledge about CD among at-risk populations and healthcare professionals, along with fear, stigma, and structural barriers significantly contribute to underdiagnosis and, consequently, to the lack of etiological treatment^11-15^, which could reduce parasitemia, severe cardiomyopathy, and mortality^16^.

In light of this, this study aims to evaluate strategies for CD diagnosis via RDTs and PCDT risk assessment questions in an endemic area.

## MATERIALS AND METHODS

### Ethics

Ethical approval was obtained from the Research Ethics Committee of the State University of Montes Claros (approval Nº 6417467). All research participants who agreed to take part in this study signed an informed consent form. Participants aged 4 to 17 years and/or those with special conditions signed an informed assent form. For all participants under 18 years of age, their legal guardian or representative signed an informed consent form prior to data collection.

### Study design

This is an analytical cross-sectional study conducted in 2023 in two municipalities, Montes Claros and Sao Joao do Pacui, in northern Minas Gerais State, Brazil. This region is considered highly endemic, with an elevated risk of vectorial transmission for CD^17^, and is among the three states most vulnerable to chronic CD in the country^18^.

### Study population and recruitment

Individuals aged one year or older, residing within the coverage area of the Family Health Strategy (FHS), were randomly invited to participate in the study. The exclusion criterion was having a previous diagnosis of CD. The sample was defined with a non-probabilistic approach.

### Data collection

Initially, contact was established with the Municipal Health Departments of the participating municipalities to ensure their approval and partnership for data collection. The data collection was conducted in October 2023 via a structured questionnaire with closed-ended questions addressing personal information and the risk criteria for *T. cruzi* infection as recommended by the PCDT. In addition to answering the questionnaire, participants also underwent capillary blood collection for a rapid test (Chagas Ab Combo Rapid Test, CTK-Biotec^®^, Poway, CA) for CD. In individuals with a positive RDT result, venous blood was collected via venipuncture for serological testing. As a control measure, for every five individuals who tested negative on the rapid test, the sixth individual in this condition was invited to undergo venous blood collection for serology.

Blood samples were centrifuged and the resulting serum was stored at −20 °C and subsequently sent on dry ice to the Central Public Health Laboratory of the State of Minas Gerais (Lacen-MG), at the Ezequiel Dias Foundation (Funed), for serological testing using two different methods—or three, when required—for CD diagnosis. The techniques used for sample analysis included enzyme-linked immunosorbent assay (ELISA), chemiluminescence immunoassay (CLIA), and indirect immunofluorescence assay (IFA). For cases in which the first two results were discrepant, a third technique, different from the initial ones, was performed. An individual was diagnosed with chronic CD if two distinct methods yielded positive results.

### Study variables

The outcome variable (dependent variable) was the serological result for Chagas disease (CD), dichotomized into two categories: Negative vs. Positive.

The RDT result was analyzed following the manufacturer’s instructions and dichotomized into two categories: Negative vs. Positive.

The individual independent variables were based on the questionnaire applied during data collection and included sex (male; female) and age, which was collected as a numerical variable and later categorized into four groups: 0–12 years, 13–17 years, 18–64 years, and over 65 years.

Regarding Chagas disease-related variables, the study considered the risk factors proposed by the PCDT for CD, assessed via the following questionnaire items: Residence in an area with CD, assessed with the question: Do you currently live or have you lived in a place where Chagas disease is present? Living in a house made of wood, wattle and daub, mud, or straw, assessed with the question: Do you currently live or have you lived in a house made of wood, wattle and daub, mud, straw, or clay? Presence of the kissing bug in the household, assessed with the question: Do you currently live or have you lived in a house where the kissing bug was present?; Blood transfusion before 1992, assessed with the question: Have you ever received a blood transfusion before 1992?; Family member with CD, assessed with the question: Does anyone in your family have Chagas disease? The response options for all these questions were: No vs. Yes.

### Statistical analyses

Initially, a descriptive analysis of all variables was conducted. Simple (n) and relative (%) frequencies were estimated for each category of variables. Bivariate analyses were performed using Pearson’s chi-square test, to analyze the association between each predictor (responses to the five questions and rapid test results) and the serology outcome. The p-value for the test was obtained via simulation to address potential issues due to low observed frequencies in some cases. Findings with *p* < 0.05 were considered statistically significant. Subsequently, an analysis of the performance of the rapid test and the five questions in diagnosing Chagas disease was conducted. In total, three strategies were considered: (i) using only the rapid test result; (ii) using only the responses to the questions; and (iii) similar to (ii), but including the rapid test result as a sixth predictor. The results are presented in terms of estimated sensitivity, specificity, and accuracy, accompanied by their respective 95% confidence intervals. Finally, for the serology and rapid test results, Cohen’s kappa coefficient was estimated to assess the agreement between the methods. All statistical analyses were performed using the R software (version 4.3.1, R Foundation for Statistical Computing, Vienna, Austria).

## RESULTS

A total of 751 individuals participated in this study, with a mean age of 51.4 (±18.2) years, ranging from 1 to 90 years. Most were female (n=434/57.8%) and resided in two municipalities in northern Minas Gerais.

**Figure 1:**
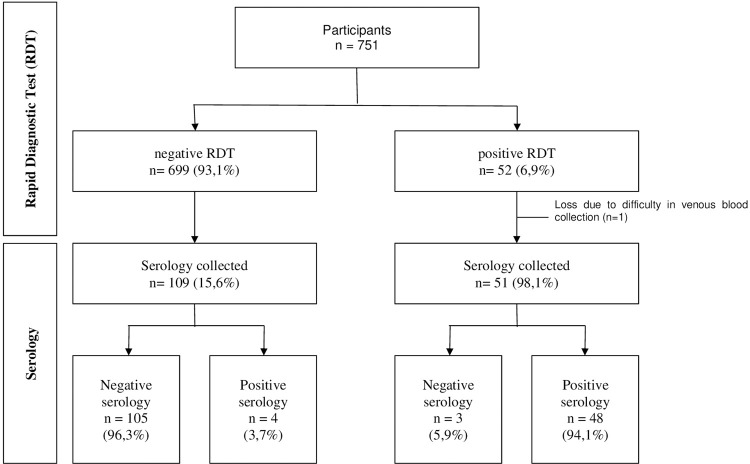
Flow of participants, Rapid Diagnostic Test (RDT) results, and serology.

All participants underwent the RDT and were questioned about risk factors. Among them, 699 (93.1%) tested negative in the RDT. Of these, 109 (15.6%) underwent serological testing, and 4 (3.7%) had a positive result. Among these four cases, follow-up data were obtained for three participants: one had previously been treated with Benznidazole, one tested negative four months later in ELISA and Hemagglutination assays, and one had a low optical density in the serological test (~S/N = 2.1). In the RDT, 52 (6.9%) participants tested positive. Among them, 51 (98.1%) underwent serological testing, and 48 (94.1%) had a confirmed positive result (Figure 1).

In the bivariate analysis conducted among those who underwent serology for Chagas disease (CD), it was observed that the RDT result, age, and risk-related questions—except for receiving a blood transfusion before 1992—were statistically associated with the outcome. Regarding serology and its association with risk factors for CD, it was found that among the individuals considered, 19 reported having none or only one risk factor, and all of them had a negative serology result. All individuals with a positive serology result reported at least two risk factors (^Table 1^).

**Table 1:** Descriptive analysis of the Rapid Diagnostic Test (RDT) result and individual characteristics and their relationship with the diagnosis of Chagas disease (n=160)

Individual variables	n (%)	Bivariate		P-value
		Negative serology for CD n (%)	Positive serology for CD n (%)	
RDT result				**<0.001**
Negative	109 (68.1)	105 (96.3)	4 (3.7)	
Positive	51 (21.9)	3 (5.9)	48 (94.1)	
Sex				0.959
Female	98 (61.3)	66 (67.3)	32 (32.7)	
Male	62 (38.8)	42 (67.7)	20 (32.3)	
Age				**0.005**
0–12 years	3 (1.9)	3 (100)	0 (0)	
13–17 years	2 (1.3)	2 (100)	0 (0)	
18–64 years	109 (68.1)	81 (74.3)	28 (25.7)	
Above 65 years	46 (28.7)	22 (47.8)	24 (52.2)	
Risk factor: lives/lived in a location with Chagas disease*				**0.001**
No	40 (25.3)	35 (87.5)	5 (12.5)	
Yes	118 (74.7)	71 (60.2)	47 (39.8)	
Risk factor: lives/lived in a wooden, wattle and daub, rammed earth, thatched, or mud house*				**0.001**
No	41 (25.8)	36 (87.8)	5 (12.2)	
Yes	118 (74.2)	71 (60.2)	47 (39.8)	
Risk factor: presence of the kissing bug in the household*				**0.001**
No	24 (15.2)	23 (95.8)	1 (4.2)	
Yes	134 (84.8)	83 (61.9)	51 (38.1)	
Risk factor: received a blood transfusion before 1992*				**0.538**
No	154 (96.9)	103 (66.9)	51 (33.1)	
Yes	5 (3.1)	4 (80.0)	1 (20)	
Risk factor: family member with CD*				**<0.001**
No	59 (37.3)	50 (84.7)	9 (15.3)	
Yes	99 (62.7)	56 (56.6)	43 (43.4)	
Number of risk factors per individual				-
0	7 (4.5)	7 (100)	0 (0)	
1	12 (7.6)	12 (100)	0 (0)	
2	28 (17.8)	24 (85.7)	4 (14.3)	
3	39 (24.8)	27 (69.2)	12 (30.8)	
4	70 (44.6)	35 (500	35 (50)	
5	1 (0.6)	0	1 (100)	

*variation of n=160.

Among the four individuals identified as false negatives, each exhibited at least two risk factors as indicated by the questionnaire (^Table 2^).

**Table 2:** Descriptive analysis of Rapid Diagnostic Test (RDT) false-negative individuals (n=4)

False-negative individuals	Lived or have lived in an area endemic for CD	Lived or have lived in a house made of wood, wattle and daub, rammed earth, thatch	Presence of Triatoma infestans (kissing bug) in the household	Received a blood transfusion before 1992	Family member with CD
Individual 1	no	yes	yes	no	no
Individual 2	yes	no	yes	no	yes
Individual 3	yes	yes	yes	no	yes
Individual 4	yes	yes	yes	no	yes

When using only the RDT, four individuals were false negatives. Considering that only 15.6% of those with a negative RDT underwent serology, it is suggested that 25 individuals (3.5%) in the entire sample may not have received a diagnosis.

^Table 3^ presents the predictive measures produced by the RDT and the responses to the five PCDT risk questions. The RDT (strategy i) provides high sensitivity (0.92; 95% CI: 0.81–0.92) and specificity (0.97; 95%CI: 0.92–0.99). Alternatively, using the five PCDT risk questions (strategy ii) yields the best sensitivity value (1.00; 95%CI: 0.93–1.00) but the lowest specificity (0.09; 95%CI: 0.05–0.17). The combined use of the RDT and the five PCDT risk questions (strategy iii) did not improve CD diagnosis compared to using the RDT alone. Finally, Cohen’s kappa coefficient was estimated for the serology and RDT results. The kappa coefficient was 0.90 (95%CI: 0.83–0.97). According to Cohen’s interpretation, kappa values above 0.80 indicate near-perfect agreement.

**Table 3:** Predictive performance of the Rapid Diagnostic Test (RDT) and risk questions for CD diagnosis

Measure	RDT % (95%CI)	Questions % (95%CI)	RDT + Questions % (95%CI)
Accuracy	96 (91; 98)	39 (32; 47)	39 (32; 47)
Sensitivity	92 (81; 98)	100 (93; 100)	100 (93; 100)
Specificity	97 (92; 99)	9 (5; 17)	9 (5; 17)

By analyzing the predictive performance considering the combination of questionnaire responses, it is observed that the measures do not surpass the use of the RDT (^Table 4^).

**Table 4:** Predictive performance of risk questions for Chagas disease diagnosis.

Measure	Positive if at least one positive response % (95%CI)	Positive if at least two positive responses % (95%CI)	Positive if at least three positive responses % (95%CI)	Positive if at least four positive responses % (95%CI)	Positive if all five responses are positive % (95%CI)
Accuracy	39 (32; 47)	45 (37; 53)	58 (50; 66)	68 (60; 75)	68 (60; 75)
Sensitivity	100 (93; 100)	100 (93; 100)	92 (81; 98)	69 (55; 81)	2 (0; 10)
Specificity	9 (5; 17)	18 (11; 27)	41 (31; 51)	67 (57; 76)	100 (97; 100)

## DISCUSSION

This study included 751 individuals from two municipalities in northern Minas Gerais, a region considered endemic for CD^19^. Among the participants, 93.1% had a negative result on the RDT, of whom 15.6% underwent serology, with confirmation in 3.7% of cases. Among the 6.9% with a positive RDT result, 98.1% underwent serology, and 94.1% of them were confirmed as positive. The RDT demonstrated superior discriminatory power, with an accuracy of 96%. The use of the risk questions proposed by the PCDT did not perform well in distinguishing CD diagnosis in an endemic area. The questionnaire demonstrates capacity to guide the diagnosis of all residents in endemic areas for serological testing, thereby enabling the identification of all individuals with CD. However, it lacks discriminatory power in endemic regions.

Early diagnosis is crucial for the control and clinical management of CD but remains difficult to access even today. In many countries, less than 10% of cases are diagnosed^9,13^. This is partly due to the fact that the disease primarily occurs in rural areas, far from major urban centers^6^. Other challenges affecting detection include centralized serological testing^1,9^ and limited knowledge and/or confidence among medical professionals in recognizing CD symptoms^12,13^. To overcome these challenges, the use of RDT^20-26^ represent a promising strategy, particularly in remote and isolated communities within endemic areas. However, despite being widely discussed in the literature, RDT is still not an established policy within Brazilian healthcare services^1^. To account for the entire local epidemiological context, strategic solutions must involve the implementation of diagnostic tools that adopt a holistic approach, including the technical training of healthcare professionals, cost-effectiveness analysis, incorporation of innovative techniques, and decentralization of serological testing in Brazil.

The findings of this study demonstrated that the use of RDT for CD diagnosis showed high accuracy (96%), sensitivity (92%), and specificity (97%). This result is similar to that of a previous study conducted in Paraguay, in which the RDT alone showed 96% agreement (κ = 0.91, 95%CI: 0.87–0.96)^26^. A study evaluating 11 different RDTs reported sensitivities ranging from 75.5% to 99.0% and specificities from 70.9% to 100%^25^. A meta-analysis of studies using RDTs for CD diagnosis found a sensitivity of 95% and specificity of 97%^27^. Recent studies comparing CD diagnostic tools observed up to 100% sensitivity in an RDT produced in Brazil^28,29^. In Brazil, the PCDT suggests using RDT as an alternative for ruling out chronic CD diagnosis, as it presents moderate evidence and weak recommendation^1^. Thus, although RDTs provide rapid and accessible results, confirmation via serological methods is still required in positive or inconclusive cases^1,20,21,23^—especially using ELISA^28-30^, which shows exceptional sensitivity (99%) and specificity (98%) for CD diagnosis^27^. The adoption of a highly sensitive method followed by a confirmatory assay in highly endemic areas, instead of using parallel testing methodologies in screening, would certainly facilitate the expansion of chronic CD diagnosis in endemic regions, improving access to diagnosis and reducing costs^31^.

The RDT shows lower specificity and sensitivity when compared to the gold standard; however, it can be considered an efficient strategy for active case detection. Its use in endemic regions should be carefully evaluated, as the risk of false negatives is high, potentially leading to missed diagnoses. The PCDT suggests using RDT in locations with limited access to analytical laboratories and for individuals with uncertain follow-up for test confirmation. RDTs may also be useful in low-demand healthcare services and in pregnant women suspected of having CD, either during prenatal care or at delivery^1^. A recent study supports this, concluding that combining RDT with conventional serology is useful for diagnostic screening in resource-constrained settings^26^.

This study identified four (3.7%) participants with false-negative RDT results. Other studies report this rate ranging from 1.02% to 5.6%, highlighting the limitations of the test in terms of diagnostic accuracy^22,25^. Although RDTs are indicated as a screening tool^20,21^, when planning active case detection strategies, it is crucial to consider that the exclusive use of RDT may lead to significant diagnostic losses. However, some studies suggest that patients with low antibody levels may represent cases of spontaneous cure^32^. It is important to note that among the four cases undetected by RDT, one had a history of previous treatment with benznidazole (BZN). In this context, RDT-based screening could aid avoid unnecessary follow-up for patients who do not require treatment or monitoring. Further studies in endemic areas are needed to assess whether patients with low antibody levels who test negative on RDT are truly at risk of developing CD.

Regarding the active case-finding strategy using only responses to the risk questions for CD proposed by the PCDT, all questions were associated with the analyzed outcome, except for the question related to blood transfusion before 1992. This result is consistent with other prevalence studies conducted in endemic regions^6,7^. This finding may be linked to the reduction in CD transmission via this route, attributed to effective screening measures implemented during blood donation^33^. Therefore, a reassessment of this specific question regarding its predictive value in endemic areas is suggested.

The use of the questionnaire demonstrated a sensitivity of 100% and a specificity of 9%. This indicates a low discriminatory power when applied in endemic areas. Although the risk questionnaire shows high sensitivity, its low specificity requires validation in non-endemic areas. In endemic regions, all individuals are likely to present at least one risk factor, compromising its diagnostic accuracy.

Thus, in endemic regions, active case-finding could forgo the additional questionnaire due to its low discriminatory power and also dispense with the use of the RDT, as the likelihood of false positives could impact patient management and treatment decisions. Adding these steps could increase the costs of active case-finding in endemic areas, as the risk questionnaire would require serological testing for all individuals, whereas RDT could lead to missed diagnoses, still requiring serology for confirmation. Although RDT could serve as a potential solution, most evaluations indicate that its sensitivity is lower than that of ELISA tests.

An important limitation of this study is related to the risk questionnaire employed, which exhibited high sensitivity due to its application in an endemic area for CD. Another limitation is the response bias associated with the questionnaire, particularly regarding the question about residing in an endemic area. This may be attributed to participants’ lack of awareness regarding the endemic status of the northern region of Minas Gerais.

However, this approach may not be as effective in non-endemic areas, where the prevalence of risk factors is significantly lower. Therefore, studies are needed to validate the sensitivity of the questionnaire in non-endemic areas, ensuring its applicability in different geographical and epidemiological contexts.

## CONCLUSION

This study assessed the accuracy of the RDT and the risk factor questionnaire for Chagas disease (CD) diagnosis proposed by the PCDT in an endemic area. The results showed that, despite the high specificity (96%) and sensitivity (92%) of the RDT, its isolated use may lead to false-negative diagnoses, and serological confirmation remains necessary. The risk questionnaire demonstrated no discriminatory power. In endemic regions, implementing RDT as a public policy in areas with access to laboratory services may not be necessary, as it would add an extra step and increase the burden on the healthcare system. Moreover, the questionnaire lacks sufficient diagnostic power, making it dispensable in endemic areas. Therefore, direct serological testing alone appears to be a sufficient diagnostic strategy in endemic areas. It is recommended that access to serological testing be strengthened by decentralizing laboratory services, training healthcare professionals, and ensuring the availability of necessary resources.

## References

[1] Brasil, Ministério da Saúde, Comissão Nacional de Incorporação de Tecnologias no SUS (2018). Diretrizes terapêuticas da doença de Chagas no âmbito do Sistema Único de Saúde.

[2] Navarro EC, Pereira PC (2013). Impact of Chagas disease on human evolution: the challenges continue. Adv Anthropol.

[3] Simões MV, Romano MM, Schmidt A, Martins KS, Marin-Neto JA (2018). Chagas disease cardiomyopathy. Int J Cardiovasc Sci.

[4] GBD 2019 Diseases and Injuries Collaborators (2020). Global burden of 369 diseases and injuries in 204 countries and territories, 1990-2019: a systematic analysis for the Global Burden of Disease Study 2019. Lancet.

[5] Martins-Melo FR, Ramos AN, Alencar CH, Heukelbach J (2014). Prevalence of Chagas disease in Brazil: systematic review and meta-analysis. Acta Trop.

[6] Cruz DS, Souza NN, Rafael AF, Damasceno RF, Ribeiro AL, Oliveira LC (2021). Serological screening for Chagas disease in an endemic region of Northern Minas Gerais, Brazil: the SaMi-Trop project. Rev Inst Med Trop Sao Paulo.

[7] Cruz DS, Damasceno RF, Leite SF, Cardoso MD, Almeida DN, Souza AB (2024). Prevalence analysis of Chagas disease by age group in an endemic region of Brazil: possible scenario of active vectorial transmission. IJID Reg.

[8] Gomes YM (1997). PCR and sero-diagnosis of chronic Chagas' disease: biotechnological advances. Appl Biochem Biotechnol.

[9] Pan American Health Organization, World Health Organization (2019). Guidelines for the diagnosis and treatment of Chagas disease.

[10] Norman FF, López-Vélez R (2019). Chagas disease: comments on the 2018 PAHO guidelines for diagnosis and management. J Travel Med.

[11] Dias JC, Ramos AN, Gontijo ED, Luquetti A, Shikanai-Yasuda MA, Coura JR (2016). II Consenso Brasileiro em Doença de Chagas, 2015. Epidemiol Serv Saude.

[12] Damasceno RF, Sabino EC, Ferreira AM, Ribeiro AL, Moreira HF, Prates TE (2020). Challenges in the care of patients with Chagas disease in the Brazilian public health system: a qualitative study with primary health care doctors. PLoS Negl Trop Dis.

[13] Ferreira AM, Sabino EC, Moreira HF, Cardoso CS, Oliveira CD, Ribeiro AL (2019). Avaliação do conhecimento acerca do manejo clínico de portadores da doença de Chagas em região endêmica no Brasil. Rev APS.

[14] Requena-Méndez A, Aldasoro E, de Lazzari E, Sicuri E, Brown M, Moore DA (2015). Prevalence of Chagas disease in Latin-American migrants living in Europe: a systematic review and meta-analysis. PLoS Negl Trop Dis.

[15] Velasco M, Gimeno-Feliú LA, Molina I, Salas-Coronas J, Solà I, Monge-Maillo B (2020). Screening for Trypanosoma cruzi infection in immigrants and refugees: systematic review and recommendations from the Spanish Society of Infectious Diseases and Clinical Microbiology. Euro Surveill.

[16] Cardoso CS, Ribeiro AL, Oliveira CD, Oliveira LC, Ferreira AM, Bierrenbach AL (2018). Beneficial effects of benznidazole in Chagas disease: NIH SaMi-Trop cohort study. PLoS Negl Trop Dis.

[17] Silveira AC, Dias JC (2011). O controle da transmissão vetorial. Rev Soc Bras Med Trop.

[18] Brasil, Ministério da Saúde, Secretaria de Vigila^ncia em Sau´de (2022). Territorialização e vulnerabilidade para doença de Chagas crônica: 14 de abril: dia mundial de combate à doença de Chagas. Bol Epidemiol.

[19] Brasil, Ministério da Saúde, Secretaria de Vigilância em Saúde e Ambiente (2024). Análise descritiva: um ano de implementação da notificação de doença de Chagas crônica no Brasil. Bol Epidemiol.

[20] Schijman AG, Alonso-Padilla J, Longhi SA, Picado A (2022). Parasitological, serological and molecular diagnosis of acute and chronic Chagas disease: from field to laboratory. Mem Inst Oswaldo Cruz.

[21] Ortega-Arroyo A, Flores-Chavez MD, Puente-Alcaraz J (2021). Combined use of two rapid tests for the conclusive diagnosis of Chagas disease: a systematic scoping review. BMJ Open.

[22] Lozano D, Rojas L, Méndez S, Casellas A, Sanz S, Ortiz L (2019). Use of rapid diagnostic tests (RDTs) for conclusive diagnosis of chronic Chagas disease: field implementation in the Bolivian Chaco region. PLoS Negl Trop Dis.

[23] Angheben A, Buonfrate D, Cruciani M, Jackson Y, Alonso-Padilla J, Gascon J (2019). Rapid immunochromatographic tests for the diagnosis of chronic Chagas disease in at-risk populations: a systematic review and meta-analysis. PLoS Negl Trop Dis.

[24] Truyens C, Dumonteil E, Alger J, Cafferata ML, Ciganda A, Gibbons L (2021). Geographic variations in test reactivity for the serological diagnosis of Trypanosoma cruzi infection. J Clin Microbiol.

[25] Marchiol A, Sanchez AC, Caicedo A, Segura M, Bautista J, Sotelo MS (2023). Laboratory evaluation of eleven rapid diagnostic tests for serological diagnosis of Chagas disease in Colombia. PLoS Negl Trop Dis.

[26] Ardiles-Ruesjas S, Lesmo V, González-Romero V, Cubilla Z, Chena L, Huber C (2025). Prevalence and diagnostic accuracy of different diagnostic tests for Chagas disease in an indigenous community of the Paraguayan Chaco. PLoS Negl Trop Dis.

[27] Suescún-Carrero S, Tadger P, Cuellar CS, Armadans-Gil L, López LX (2022). Rapid diagnostic tests and ELISA for diagnosing chronic Chagas disease: systematic revision and meta-analysis. PLoS Negl Trop Dis.

[28] Ostermayer AL, Medeiros FA, Iturra JA, Souza JA, Leony LM, Vasconcelos LC (2025). A multicentre comparative study of serological methods for diagnosing Chagas disease in Brazil. Mem Inst Oswaldo Cruz.

[29] Iturra JA, Leony LM, Medeiros FA, Souza JA, Siriano LR, Tavares SB (2023). A multicenter comparative study of the performance of four rapid immunochromatographic tests for the detection of anti-Trypanosoma cruzi antibodies in Brazil. Front Med (Lausanne).

[30] Ascanio LC, Carroll S, Paniz-Mondolfi A, Ramírez JD (2024). In vitro diagnostic methods of Chagas disease in the clinical laboratory: a scoping review. Front Microbiol.

[31] Sabino EC, Nunes MC, Blum J, Molina I, Ribeiro AL (2024). Cardiac involvement in Chagas disease and African trypanosomiasis. Nat Rev Cardiol.

[32] Remesar MC, Ester EC, Buss LF, Merlo CD, López MG, Humeres SL (2025). Bimodal distributions of anti-Trypanosoma cruzi antibody levels in blood donors are associated with parasite detection and antibody waning in peripheral blood. PLoS Negl Trop Dis.

[33] Pati I, Cruciani M, Masiello F, Barone F, Silvioli G, La Raja M (2022). Chagas disease and transfusion risk in Italy: the results of a national survey. Pathogens.

